# Supplementary material for: Too much to handle? Interference from distractors with similar affordances on target selection for handled objects
Source: PLoS One. 2023 Aug 29;18(8):e0290226. doi: 10.1371/journal.pone.0290226 (PMC10464981; doi:10.1371/journal.pone.0290226)
Supplement: S1 Appendix — (DOCX) [file pone.0290226.s001.docx]

**S1 APPENDIX: List of objects pairs**

| **Kitchen utensil** | **Tool** |
| --- | --- |
| Dish brush | Screwdriver |
| Nutcracker | Tool file |
| Pan | Rake |
| Bottle opener | Shovel |
| Whisk | Paint roller |
| Ladle | Brush |
| Mug | Paint brush |
| Strainer | Squeegee |
| Tea ball | Trowel |
| Rolling pin | Hoe |
| Spatula | Wrench |
| Teapot | Hammer |
| *Pasta server* | *Iron* |

The example appears in *Italic*
