# Supplementary material for: Too much to handle? Interference from distractors with similar affordances on target selection for handled objects
Source: PLoS One. 2023 Aug 29;18(8):e0290226. doi: 10.1371/journal.pone.0290226 (PMC10464981; doi:10.1371/journal.pone.0290226)
Supplement: S2 Appendix — (DOCX) [file pone.0290226.s002.docx]

**S2 APPENDIX: Mixed models structures and R syntaxes**

**Accuracy:**

Mixed model of Experiment 1:

ACC = Similarity * Compatibility*Response hand

+ (1 | Participants : Task Version)

+ (1 | Items)

Mixed model Experiment 2:

ACC = Similarity * Compatibility*Response hand

+ (1 | Items)

**Response times:**

Experiment 1

RT = Similarity * Compatibility*Response hand

+ (1+ Similarity + Compatibility+ Response hand | Participants : Task version)

+ (1 | Items)

Experiment 2:

RT = Similarity * Compatibility*Response hand

+ (1 + Response hand | Participants : Task version)

+ (1 | Items)

Merged Experiments 1 and 2:

RT = Similarity * Compatibility*Response hand*Experiment Version

+ (1+ Compatibility+ Response hand | Participants : Task version : Experiment)

+ (1 | Items)

Right hand for merged Experiments 1 and 2:

RT = Similarity * Compatibility

+ (1+ Compatibility+ Response Hand | Participants : Task version : Experiment Version)

+ (1 | Items)

Left hand merged Experiments 1 and 2:

RT = Similarity + Compatibility

+ Similarity *Target-Response Compatibility

+ (1+ Compatibility | Participants : Task version : Experiment Version)

+ (1 | Items)

**Complementary analysis on compatibility effects:**

Merged Experiments 1 and 2:

Compatibility Effect = Similarity *Response hand*Experiment Version

+ (1 | Participants : Task version : Experiment)
