# Supplementary material for: Too much to handle? Interference from distractors with similar affordances on target selection for handled objects
Source: PLoS One. 2023 Aug 29;18(8):e0290226. doi: 10.1371/journal.pone.0290226 (PMC10464981; doi:10.1371/journal.pone.0290226)
Supplement: S3 Appendix — (DOCX) [file pone.0290226.s003.docx]

**S3 APPENDIX: Tables**

Experiment 1, mean (SD) RTs in milliseconds:

| **Response hand** | **Similarity** | **Compatibility** | **Mean (SD)** |
| --- | --- | --- | --- |
| Right hand | Similar | Compatible | 895.110 (139.219) |
|  |  | Incompatible | 889.576 (145.151) |
|  | Dissimilar | Compatible | 878.637 (141.185) |
|  |  | Incompatible | 895.005 (153.863) |
| Left hand | Similar | Compatible | 865.831 (154.127) |
|  |  | Incompatible | 872.197 (135.851) |
|  | Dissimilar | Compatible | 867.585 (137.864) |
|  |  | Incompatible | 873.028 (153.788) |

Experiment 2, mean (SD) RTs in milliseconds:

| **Response hand** | **Similarity** | **Compatibility** | **Mean (SD)** |
| --- | --- | --- | --- |
| Right hand | Similar | Compatible | 910.594 (145.733) |
|  |  | Incompatible | 906.501 (144.945) |
|  | Dissimilar | Compatible | 896.696 (141.465) |
|  |  | Incompatible | 912.204 (150.342) |
| Left hand | Similar | Compatible | 883.353 (148.043) |
|  |  | Incompatible | 886.363 (142.500) |
|  | Dissimilar | Compatible | 886.197 (160.577) |
|  |  | Incompatible | 888.333 (154.548) |

Combined data of experiments 1 and 2, mean (SD) RTs in milliseconds:

| **Response hand** | **Similarity** | **Compatibility** | **Mean (SD)** |
| --- | --- | --- | --- |
| Right hand | Similar | Compatible | 901.231 (142.135) |
|  |  | Incompatible | 895.690 (142.865) |
|  | Dissimilar | Compatible | 885.950 (140.974) |
|  |  | Incompatible | 901.519 (151.591) |
| Left hand | Similar | Compatible | 873.668 (151.769) |
|  |  | Incompatible | 877.717 (138.688) |
|  | Dissimilar | Compatible | 875.646 (149.066) |
|  |  | Incompatible | 879.423 (154.397) |
